# The impact of the decomposition process of shallow graves on soil mite abundance

**DOI:** 10.1111/1556-4029.14906

**Published:** 2021-10-14

**Authors:** Jas K. Rai, Brian J. Pickles, M. Alejandra Perotti

**Affiliations:** ^1^ Ecology and Evolutionary Biology Section School of Biological Sciences Health and Life Sciences Building University of Reading Reading UK

**Keywords:** abiotic, acarines, decay stage, edaphic, forensic acarology, shallow grave, soil pH, *Sus scrofa domesticus*

## Abstract

Burial of a cadaver results in a slower decomposition rate, due to more stable below‐ground temperatures and restricted access to necrophagous insects. In such circumstances, analysis of the soil mesofauna, with emphasis on mites (Acari) may be more valuable in time‐of‐death estimations. The production of volatile organic compounds of cadaveric decay results in changes, especially in the soil pH, which in turn would affect the abundance and diversity of the associated mites. In general, the effects of decomposition and the consequently altered pH levels on the abundance of mites in shallow graves, as well as the effects of fluctuating above‐ground environmental parameters (temperature, relative humidity, and precipitation) remain unknown. Here, we found that the decay of three pig cadavers buried in shallow graves (<30 cm below) caused a significant increase in the soil pH throughout decomposition, from neutral to alkaline. Cadaver decay attracted an abundance of mites: with 300 mites collected from the three pig cadavers compared to 129 from the control soil samples at the same depth. Mites rapidly became more abundant in cadaver‐associated soils than in control soils after the fresh stage. Increasing soil pH had a positive impact on the abundance of mites in graves and there was a significant interaction between cadaver body temperature and soil pH. Above‐ground fluctuations in temperature, relative humidity, and precipitation were found to have no significant direct effect on mite abundance in grave or control soils.

**Highlights**

- Cadavers buried in shallow graves cause an increase in soil pH.
- Cadaver temperatures in shallow graves are closely influenced by the above‐ground temperatures.
- Mites colonize and rapidly increase in abundance between fresh and dry decay.
- Temperature, humidity, rain, and snow have no significant impact on mite abundance in shallow graves.
- Soil pH is the major environmental factor impacting mite abundance in shallow graves.

## 1 INTRODUCTION

Decomposition of exposed and buried cadavers is categorized into a series of stages of decay based on changes in physical characteristics [1, 2, 3, 4, 5]. A handful of studies have described the stages of decay of buried cadavers. Payne et al. [2] described five decay stages for buried pigs as fresh, inflated, deflation and decomposition, disintegration, and skeletonization. Vanlaerhoven and Anderson [5] also described five stages of decay of pigs buried at 30 cm: fresh, bloat, active, advanced, and dry/remains. Typically, these five stages are most widely described in vertebrate decomposition studies of both exposed and buried cadavers [8].

While the process of decomposition of buried and exposed cadavers is the same, the rate of decomposition of buried cadavers is much slower [5, 6, 7, 8, 9]. The biotic and abiotic parameters below ground are different to those above ground and the reduced insect activity in a grave environment means that buried bodies will decompose at an entirely different rate compared to an exposed cadaver in the same locality [10]. Temperature, access to insects, and the depth of burial are considered to be the three most influential factors [11]. Hence, decomposition of buried cadavers can be more difficult to characterize as the burial, depending on the depth of the grave, may significantly influence the temperature in the headspace of the corpse as well as the level of access to insects.

In terrestrial environments, a decomposing cadaver forms a nutrient‐rich substrate for a variety of necrophagous, predatory/parasitic, omnivorous, and adventive arthropods. Each decay stage attracts a unique assemblage of arthropods which arrive at the carcass in a series of waves, resulting in faunal succession occurring in a relatively predictable pattern [6, 12]. The majority of existing research of the grave fauna is limited to insects, and that of other soil arthropods is largely unexamined. Several centimeters of soil covering the carcass may prevent access to a cadaver for major Calliphoridae species (*Calliphora* and *Lucilia*) [13, 14, 15]. Some insects can still gain access to an interred cadaver through macro‐pores in the soil, though the access is restricted depending on the depth of burial and pore size [5, 9, 10, 15, 16, 17, 18]. There is also limited knowledge on how far insects travel to colonize buried carcasses and only a handful of dipteran species (and some Coleoptera) are considered to have forensic importance in graves [15, 19, 20, 21]. For example, the Phoridae species *Conicera tibialis* [22, 23, 24, 25] and *Megaselia scalaris* are frequently sampled from graves and utilized in PMI estimations [26, 27].

The occurrence of insects in graves is somewhat random due to their limited access, so the use of insect succession in PMI estimations and trace evidence analysis of buried bodies can become unreliable. In circumstances, where activities and visitation patterns of insects are reduced or altered, analysis of the soil mesofauna, particularly the composition of the most predominant taxa, mites (Acari) inhabiting the surrounding soil may hold the forensic value. Several studies have demonstrated that mites are an important part of the cadaver fauna throughout decomposition [5, 28, 29, 30, 31, 32]. However, the variations in abundance and diversity of mites associated with each decomposing stage of buried cadavers in terrestrial habitats have not been explored. Mites are ubiquitous in natural and synanthropic habitats and are the most species‐rich and abundant soil micro‐arthropods in most soil types [33]. Mites have diverse feeding strategies and in soil, habitats are major contributors of organic decomposition of vertebrate tissue and dead plant matter. Mites, therefore, form a large part of the cadaveric fauna, especially in graves, where insect activity is absent or significantly reduced. More than 250,000 mites may be found in 10‐cm depth in a square meter of forest soil and they are especially numerous in upper horizons where oribatids dominate, as compared to much greater depths, where mesostigmatids, astigmatids, and prostigmatids are predominant [34, 35].

Mite population densities are affected by the fluctuations in the physical and chemical properties of soil [36]. During mammalian decomposition, the leakage of nutrient‐rich cadaver fluids contributes to the alteration of the micro‐environment and chemistry of the soil, in particular the soil pH, which in turn impacts the meso‐faunal organisms. In several studies, cadaver decay has been found to cause an increase in the soil pH from the start to mid decomposition, to a more alkaline level, followed by a decrease toward the end stages of decay [37, 38, 39, 40]. This is attributed mainly to the production and accumulation of ammonium ions (${\text{NH}}_{4}^{+}$) from the ammonification of proteins during autolysis and putrefaction occurring mainly during bloated and active decay [38, 41]. The changes in soil pH are thought to directly affect the abundance of the meso‐fauna throughout the decomposition, in particular the mites [42], however, the effect of the alteration in soil pH during decay on the abundance of the surrounding soil micro‐arthropods such as mites is poorly understood.

The fauna of a cadaver is closely associated with the decomposition stages. However, the fauna may also be influenced by several extrinsic factors that are not directly linked to the cadaver or circumstances surrounding the death, such as the climatic conditions, ambient temperature, ambient humidity, rainfall, and snowfall. These factors along with the intrinsic factors such as changes in the soil pH (as a result of decomposition) can significantly influence the fauna associated in terms of abundance and diversity. When a body is buried in a grave, it has greater protection from fluctuating environmental conditions such as rain, temperature, humidity, and snow, however, a grave of less than 20‐cm depth is likely to be affected by the fluctuations in the above‐ground environmental conditions [43]. The deeper the grave, the more protected the cadaver and its associated fauna are from environmental instabilities. Exposed cadavers, in terms of decomposition rate and the colonizing fauna, are more sensitive to such environmental parameters [5, 10]. For example, parameters such as rain, snow, and ambient temperature can have a significant effect on the presence and abundance of fauna such as Diptera colonizing an exposed cadaver outdoors [44]. Calliphoridae activity is reduced during higher periods of rain and snowfall, and rainfall, in particular, can delay oviposition and pupation [26, 45], while higher temperatures are more favorable for oviposition and development of some necrophagous flies and beetles [26]. It is not known if the mite fauna within a grave environment, where the cadaver is concealed by a thin layer of soil such as that in a shallow grave of less than 30 cm, will be affected by external factors such as temperature, humidity, rain, and snowfall in the same way that an exposed cadaver may experience.

Changes in the soil pH levels around a decaying cadaver have a major impact on the biotic elements, such as the population densities of bacteria, fungi, and mites [46, 47]. For instance, neutral to slightly alkaline pH levels are favorable for soil bacteria, while acidic pH levels tend to be more favorable for fungal growth [46]. In turn, this impacts the composition of micro‐arthropods such as mites that feed on these soil organisms [48]. The soil pH levels also influence abiotic parameters such as carbon content and nutrient availability, which fluctuate in response to cadaveric decay and directly influence the surrounding soil fauna [37, 38]. However, the patterns of mite abundance associated with fluctuating soil pH during cadaveric decay are mostly unknown. Other extrinsic factors, such as seasonal fluctuations of temperature and rainfall, also impact mite densities [49, 50], yet under cadaver decay circumstances, it is not known if these factors have a greater or a lesser effect on the abundance of mites that the effects of decomposition itself (food, shelter availability and soil pH).

### 1.1 Aims and objectives

To use entomological and acarological evidence for time‐of‐death estimations, it is crucial to understand the stages and rates of decomposition that a cadaver will undergo in various scenarios and circumstances of death. The stage of decomposition will determine the abundance and diversity of mites in the surrounding soil, which is thought mainly to be an effect of food availability, though abiotic factors may also contribute. However, the extent to which fluctuations in soil pH, ambient temperature, humidity, and precipitation affect the mite abundances within a shallow grave environment remains unknown. Here, we examined the decomposition of pig cadavers in shallow graves to: (1) examine the patterns of total mite abundance associated with each decomposition stage, (2) investigate any links between the environmental factors and the decay of buried cadavers, and (3) analyze whether soil pH, ambient temperature, ambient relative humidity, and daily rainfall are associated with the abundance of mites throughout the decomposition process.

## 2 METHODS

### 2.1 Experimental design

The study was carried out over a period of 3 years (October 3, 2015 to September 30, 2018) within a temperate woodland located in the grounds of the Whiteknights Campus, University of Reading, Berkshire, UK (51°26′10.6″N 0°56′35.0″W). The relatively undisturbed area of deciduous trees and shrubbery measured approximately 80 m × 50 m. Although the main road is adjacent to the site, it is well concealed from the public view with the thick vegetation. The soil in this region is clay loam soil and slightly acidic with an average pH of 6.9.

Three freshly killed pig carcasses (*Sus scrofa domesticus*) (mean 33 kg) purchased from a slaughterhouse were used as temporal repeats (one per year), representing suitable proxies for the decay of human remains [12, 51]. Each pig was killed on the day that each study commenced, every year. The total depth of the grave (10–22 cm) varied based on the carcass size as each carcass naturally varied in volume, and all carcasses were covered with 3–4 cm of top soil. Burial took place in the first half of October each year, with each study period ending when only bones remained. Each cadaver was placed in a body bag immediately postdeath and transported to the study site and buried within 3–6 h. Consecutive grave plots (P1, P2, P3) were located at a minimum of 10 m from the previous grave plot with control soil plots (C1, C2, C3) located at a minimum of 20 m from graves and a minimum of 10 m from the previous control plots. A metal wire cage was used to protect the carcasses from the vertebrate scavengers while allowing access to invertebrate scavengers. Use and transport of carcasses followed the regulations established by the Animal Health and Veterinary Laboratories Agency (AHVLA), after registration: *ABP Registration Reference: U1116918 (Notification of registration for the generation, transportation, handling, processing, storage, placing on the market, distribution, use or disposal of animal by‐products (ABP's), or derived products under the requirements of Article 23 of Regulation (EC) No. 1069/2009)*.

### 2.2 Soil sampling and mite extraction

Digital images were taken to record the decomposition process of all three cadavers. The decay process of each carcass was grouped into five major decomposition stages, which were established by the observations of physical post‐mortem changes to the cadavers. ‘Cadaver soils’ were collected as follows: A total of‐300 ml volume of soil was sampled from immediately beneath each of the head, abdomen, and posterior regions of the pig carcass (total = 900 ml soil per sampling day) by lifting it at an angle with a large shovel and collecting soil from underneath with a hand shovel. Each carcass was inspected 3–5 times per week to record post‐mortem changes (e.g., odors, maggot activity), and sampling days were selected corresponding to the five stages of decomposition (determined by the observation of the physical post‐mortem changes). Sampling took place thrice within each stage. ‘Control soils’ were collected on the same day from the corresponding control plot, with three samples of 300‐ml soil taken from the same depth as the cadaver soils.

Mite extraction was conducted for each sample over 7 days using manually constructed Berlese‐Tullgren funnels placed underneath 40‐watt incandescent light bulbs. Here, arthropods travel downward to avoid heat and light (Tullgren, 1918) where they are collected in vials of 70% (v/v) ethanol, preserving them prior to counting. Collection vial contents were transferred into petri dishes where a stereomicroscope was used to separate mites from all other arthropods, with any phoretic mites manually removed from each of these arthropods. Mites were then counted to generate the total for each soil sample (*n* = 90), corresponding to one of the five stages of decomposition for a cadaver soil (*n* = 45) or a corresponding control soil (*n* = 45).

### 2.3 Collection of environmental data

Environmental data for each sampling day over the period October 01, 2015 to September 30, 2018 were retrieved from the Department of Meteorology weather station at the University of Reading, Whiteknights campus, UK, which is located approximately 600 m from the study site. The data included Dry bulb temperature (DBT) (°C) and relative humidity (RH) (%), recorded every hour over 24 h, and the total daily rainfall (mm), and total daily snow depth (cm) starting at 0900 GMT. The highest average monthly temperature over the 3 years was during July 2017 and August 2018 (31.2°C), while the lowest was during February 2018 (3.1°C). The highest average monthly humidity was during September 2017 (97.0%), while the lowest was seen during July 2018 (60.6%). Rainfall occurred during every month of the study. The highest average monthly rainfall occurred during July 2017 (4 mm), while the lowest occurred during June 2018 (0.2 mm). The average monthly snowfall was 0 cm for every month except during March 2018. Only a single sampling day was associated with the snowfall (1 cm); so, these data were not studied further.

The body surface temperature (°C) of each pig carcass was recorded using a handheld digital infra‐red thermometer (Tekpower infrared DT8380 thermometer). Temperatures of the surface of the head, the torso, and the posterior region of the carcasses were taken on each day the soil was sampled and were averaged to give the mean body surface temperature. To obtain the pH of each sample of cadaver and control soil, 10 g of soil was mixed in 25 ml of deionized water following the soil pH measurement ratio of 1:2.5 (soil: solution) suggested by Aciego Pietri and Brookes [52]. After settling at room temperature for 30 min, a pen‐type pH meter (Tekco plus Digital pH meter) was used to record the pH of the soil solution.

### 2.4 Statistical analyses

For all the statistical tests, a *p*‐value < 0.05 was accepted for rejecting the null hypothesis. Data on the duration of decomposition were tested for normality using the Anderson‐Darling test. To examine the differences in the duration of the five major decomposition stages of the buried cadavers, a one‐way analysis of variance (ANOVA) with post hoc Tukey honest significant difference (HSD) pairwise comparison was conducted with Minitab^®^ 19.2020.1.0.

Differences between cadaver body surface temperature and ambient air temperature were tested for significance using non‐parametric Mann–Whitney *U*‐tests in Minitab^®^ 19.2020.1.0.

Statistical analyses of factors influencing mite abundance were conducted in R version 4.0.2 [53]. Generalized linear models with a Poisson distribution were initially used to examine the data. Due to the overdispersion of the data, generalized linear mixed‐effects models (GLMM) with a negative binomial error structure and ‘replicate’ as a random factor were constructed to examine the changes in the mite abundance using the R package ‘lme4’ [54]. Models were fitted by restricted maximum likelihood. Interaction terms were examined for the variance‐inflation due to multicollinearity using the *vif* function (VIF, variance‐inflation factors) in R package ‘car’ [55]. Model fit for negative binomial GLMs was estimated using marginal ($R_{\left(m\right)}^{2}$; variance explained by only the fixed effects) and conditional ($R_{\left(c\right)}^{2}$; variance explained by the entire model, including both fixed and random effects) values with the trigamma method following [56].

## 3 RESULTS

### 3.1 Timeline of cadaver decomposition

**FIGURE 1:**
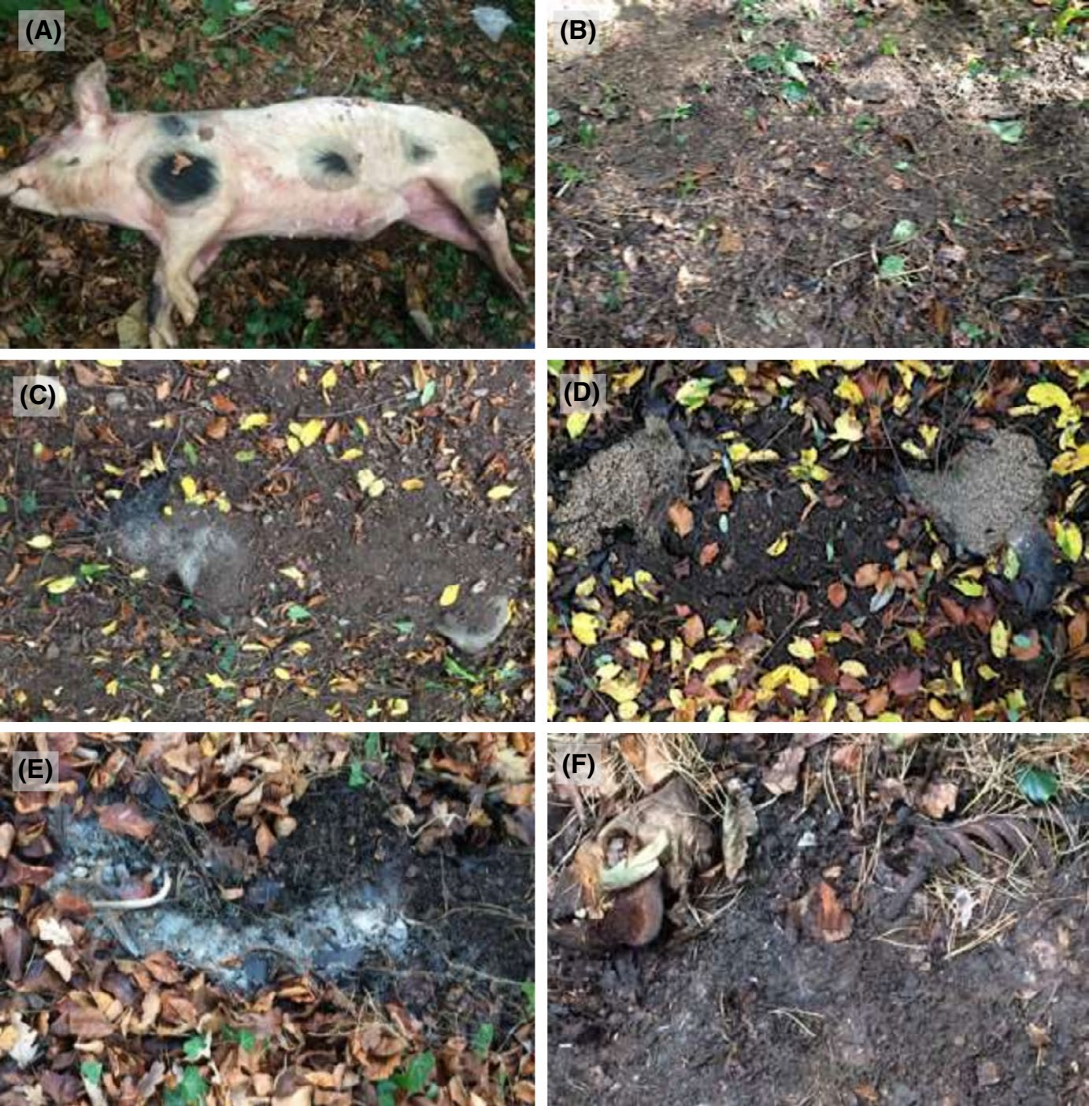
The five major stages of decomposition (fresh, bloated, active, advanced, and dry/remains), cadaver P3 are shown as reference (A) Fresh before burial, (B) after burial concealed with soil, (C) bloated showing areas of skin exposure, (D) active, (E) advanced, and (F) dry/remains [Color figure can be viewed at wileyonlinelibrary.com]

**FIGURE 2:**
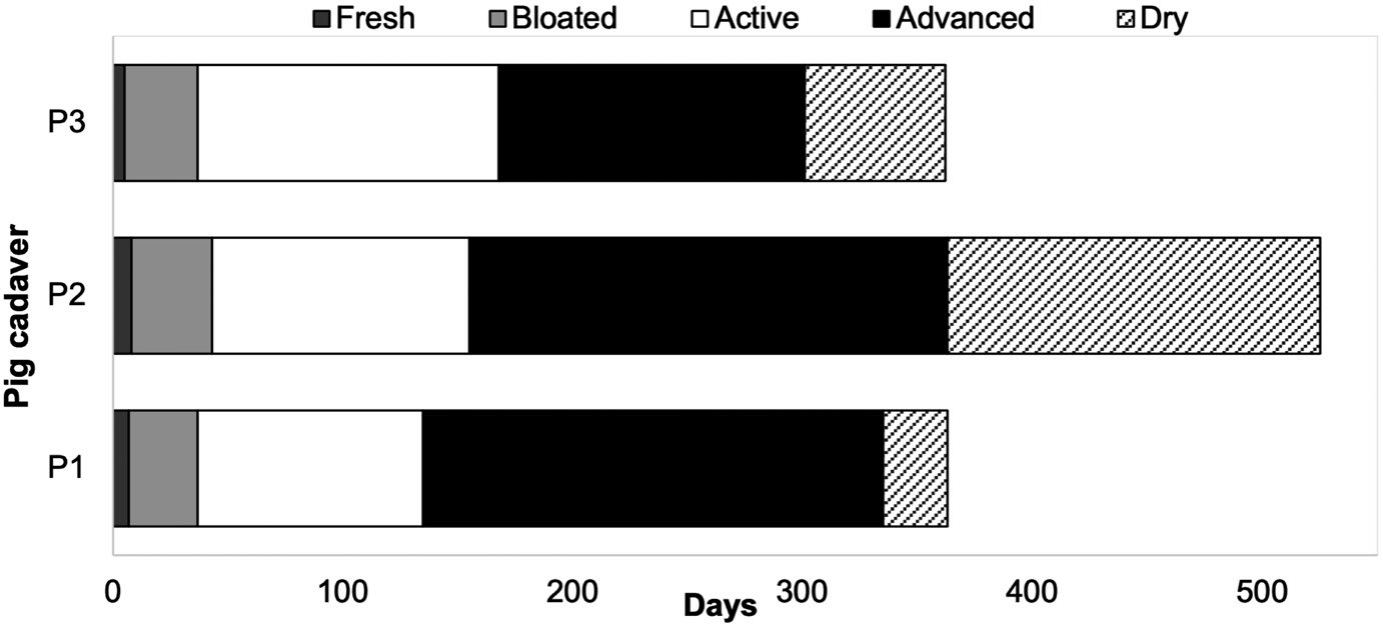
The duration of the five major stages of decomposition (fresh, bloated, active, advanced, and dry/remains) of each buried pig cadaver (P1, P2, P3)

All three pig cadavers underwent the expected five major stages of decomposition for buried and exposed cadavers: fresh, bloated, active, advanced, and dry/remains (Figure 1). The decomposition process of all three cadavers from the fresh stage to the dry stage lasted over a year (Figure 2). From the fresh stage at day 1, the onset of the final stage (dry/remains) was evident for P1 by skeletonization of the skull and torso at day 336, for P2 at day 364, and for P3 at day 302 (Figure 1).

The initial post‐mortem changes that were evident during the fresh stage of the three cadavers included: (i) algor mortis, which occurred gradually within 24 h post‐mortem of all three cadavers, (ii) rigor mortis, which took place for all three cadavers between 4 and 6 h post‐mortem, and (iii) livor mortis, which was evident as purple discoloration of the lower parts of the body approximately 4–6 h post‐mortem. The three carcasses also entered the bloated stage at a similar time: onset of bloating in P1 was evident on day 8, lasting 30 days, in P2 on day 9, lasting 35 days, and in P3 on day 6, lasting 32 days. Exposure of parts of the carcass was caused by the natural inflation of the carcass during the bloated stage. As the cadavers distended mainly around the abdominal region, parts of the loose top‐soil covering the cadavers were pushed away from some regions resulting in small areas of the cadavers exposed from the shallow graves. This allowed for small areas of access to the cadavers for dipterans to begin colonization (Figure 1C). Decomposition odors at this stage were prominent and, in all subjects, Diptera was seen visiting the cadavers, with initial ovipositing on the exposed body surface observed.

P1 and P3 transitioned into active decay 38 days post‐mortem, lasting 98 days and 112 days, respectively, whereas for P2, the initial signs of active decay (such as purging of decay fluids) were evident slightly later (day 44) and lasted longer (131 days). The active stage was characterized in all cadavers by visible deflation of the carcasses (sinking of the grave surface), liquefaction of soft tissue, and purging of decay fluids, which caused the soil to become heavily saturated with decomposition fluids. Active decay was also marked by Dipteran larval masses causing most of the soft tissue to break down and the formation of pungent decomposition odors (Figure 1D). Larvae were mainly visible on exposed cadaver regions, for example, larval masses were prominent around the head and posterior regions of P3 from day 38.

Advanced decomposition was the longest decay stage for all three cadavers and was marked by the degradation of most of the soft tissue, with some dried soft tissue and remnants of decay fluids present, but mostly skin, cartilage, bone remaining, and weak decomposition odors (Figure 1E). There was a reduction in decay fluids in the soil, resulting in the further sinking of the grave surface, and Diptera larvae were absent. The onset of advanced decay in P1 was evident on day 136, lasting 200 days, in P2 on day 156, lasting 208 days, and in P3 on day 169, lasting 133 days.

The final stage of decomposition, dry/remains, was characterized by the presence of dry skin, bones, and hair, desiccation of the surrounding soil, reduced arthropod activity, and minimal decomposition odors (Figure 1E). The skeletonization stage of buried cadavers can last a relatively long period of time with no further significant post‐mortem changes occurring for several months to years. The dry/remains stages of all the three cadavers were studied from the initial skeletonization of the body (day 336 for P1, day 364 for P2 and day 302 for P3) until no further dehydration of the carcass and the surrounding soil was evident, which was on day 364 for P1, day 526 for P2 and day 363 for P3.

The duration data of all five decomposition stages (Table 1) was normally distributed according to the Anderson‐Darling test for normality (*p* > 0.05). One‐way ANOVA with post hoc Tukey HSD showed that there was a significant difference in the duration between the decomposition stages of the buried cadavers (*F*_df 4,10_ = 10.29; *p* = 0.001; factor contribution = 80.45%, error contribution = 19.55%). The pairwise comparisons (Table 2) showed that the fresh stage, which was the shortest for all three cadavers (mean 6.7, standard deviation [SD] 1.53), was significantly shorter than the active (mean 113.67, SD 16.56) (*p* < 0.05) and the advanced decomposition (mean 180.3, SD 41.2) (*p* < 0.05). The advanced stage was the longest for all the three cadavers and was significantly longer than both the fresh stage and the bloated stage (mean 32.33, SD 2.52) (*p* < 0.05). The dry stage of decomposition (mean 83.7, SD 69.8) was not significantly different in the duration compared to the advanced stage at the threshold selected for the test (*p* = 0.06), which was likely due to the unusually long dry stage observed in P2 (Tables 1 and 2).

**TABLE 1:** Mean, standard deviation (SD), minimum, and maximum duration of decomposition stages of buried pig cadavers

Stage of decomposition	Mean	SD	Minimum (days)	Maximum (days)
Fresh	6.67	1.53	5	8
Bloated	32.33	2.52	30	35
Active	113.67	16.56	98	131
Advanced	180.30	41.20	133	208
Dry	83.70	69.80	28	162

**TABLE 2:** Pairwise comparisons of decomposition stage durations using post hoc Tukey HSD simultaneous tests with a significant differences (*p* < 0.05) indicated in bold

Difference of levels	Difference of means	SE of difference	95% CI	*T*‐value	Adjusted *p*‐value
Bloated ‐ Fresh	25.7	30.2	−73.7, 125.1	0.85	0.909
Active ‐ Fresh	107.0	30.2	7.6, 206.4	3.54	**0.034**
Advanced ‐ Fresh	173.7	30.2	74.3, 273.1	5.74	**0.001**
Dry ‐ Fresh	77.0	30.2	−22.4, 176.4	2.55	0.155
Active ‐ Bloated	81.3	30.2	−18.1, 180.7	2.69	0.126
Advanced ‐ Bloated	148.0	30.2	48.6, 247.4	4.90	**0.004**
Dry ‐ Bloated	51.3	30.2	−48.1, 150.7	1.70	0.476
Advanced ‐ Active	66.7	30.2	−32.7, 166.1	2.21	0.253
Dry ‐ Active	−30.0	30.2	−129.4, 69.4	−0.99	0.853
Dry ‐ Advanced	−96.7	30.2	−196.1, 2.7	−3.20	0.058

### 3.2 Changes in mite abundance with decay stage

**FIGURE 3:**
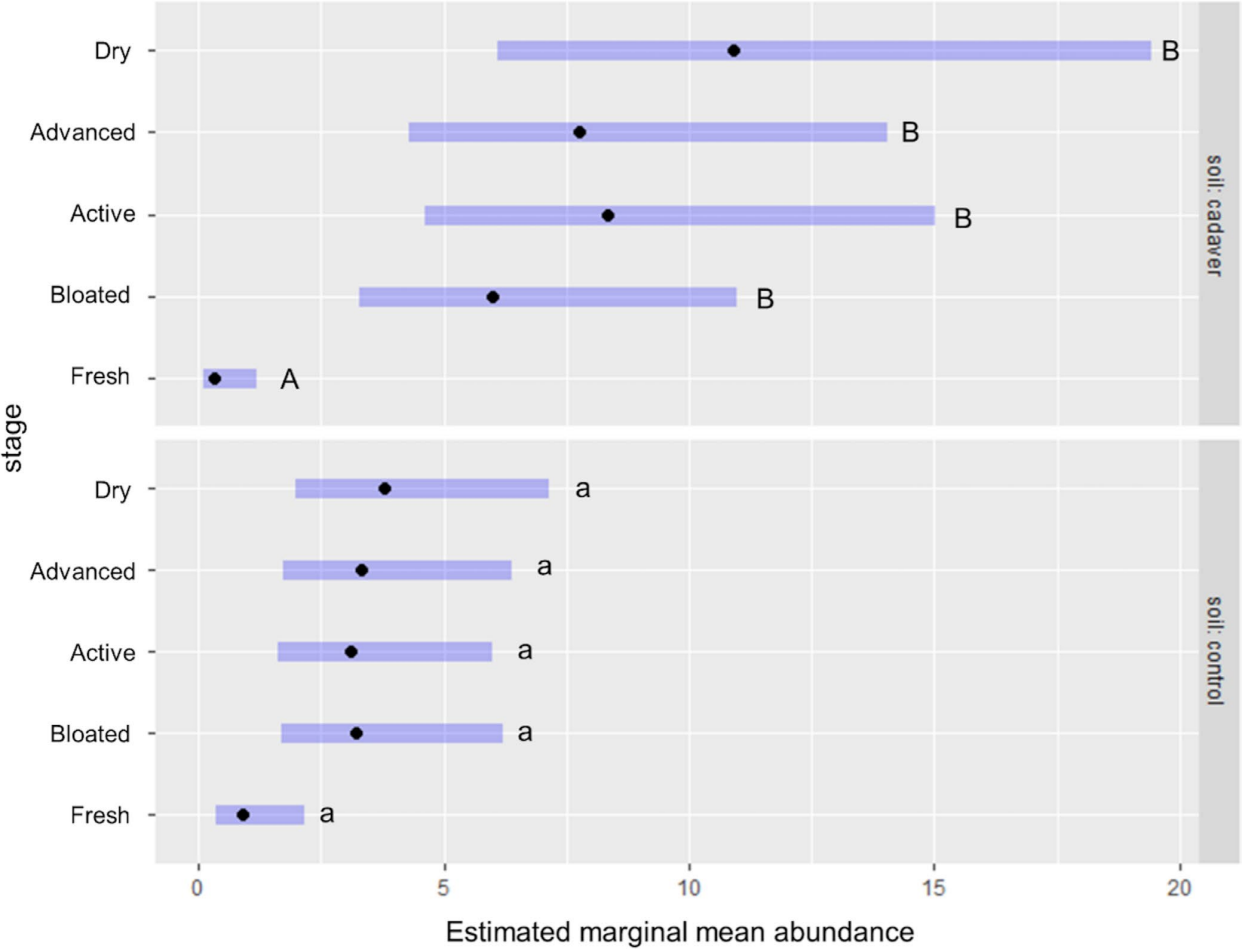
Mite abundance within cadaver and control soils at each decay stage using estimated marginal means. Different letters indicate significant differences based on Tukey's HSD (performed on the log scale). Blue bars = 95% confidence intervals [Color figure can be viewed at wileyonlinelibrary.com]

A total of 300 mites were recovered from the grave soil of all three cadavers (Median 103, Interquartile range [IQR] 27), while a total of 129 mites were collected from the control soils (Median 45, IQR 58). The abundance of mites was found to vary significantly with decomposition stage (fresh, bloated, active, advanced, dry), soil treatment (cadaver or control), and their interaction (Table 3, Figure 3, Table S1). The estimated model fit ($R_{\left(m\right)}^{2}$ = 0.425|$R_{\left(c\right)}^{2}$ = 0.425) indicated that decomposition stage and soil treatment, rather than differences between replicates, were responsible for the observed variation in mite abundance. The overall pattern revealed a significant increase in mite abundance in cadaver soils during the bloated, active, advanced, and dry stages compared to the fresh stage (*p* < 0.001). Mite abundance in control soils was not significantly different from that of cadaver soils during the fresh (*p* = 0.210) or bloated (*p* = 0.077) stages, but was significantly lower in control compared to cadaver soils during the active, advanced, and dry stages (*p* < 0.05) (Table 3; Figure 3).

**TABLE 3:** Results of negative binomial GLMM examining changes in the abundance of mites between decomposition stages, soil types, and their interaction (baseline = fresh Cadaver soil)

	Estimate	Std.error	*Z* value	*p* (>[*z*])
Intercept	−1.099	0.641	−1.715	0.086
Bloated stage	2.890	0.711	4.065	**4.81e‐05**
Active stage	3.219	0.707	4.550	**5.36e‐06**
Advanced stage	3.150	0.708	4.448	**8.65e‐06**
Dry stage	3.486	0.705	4.944	**7.66e‐07**
Control soil	0.981	0.782	1.254	0.210
Bloated:Control	−1.603	0.905	−1.771	0.077
Active:Control	−1.966	0.903	−2.178	**0.029**
Advanced:Control	−1.828	0.902	−2.027	**0.043**
Dry:Control	−2.039	0.898	−2.272	**0.023**

Note

In bold, significantly different *p* values.

### 3.3 Influence of environment on cadaver decay

**FIGURE 4:**
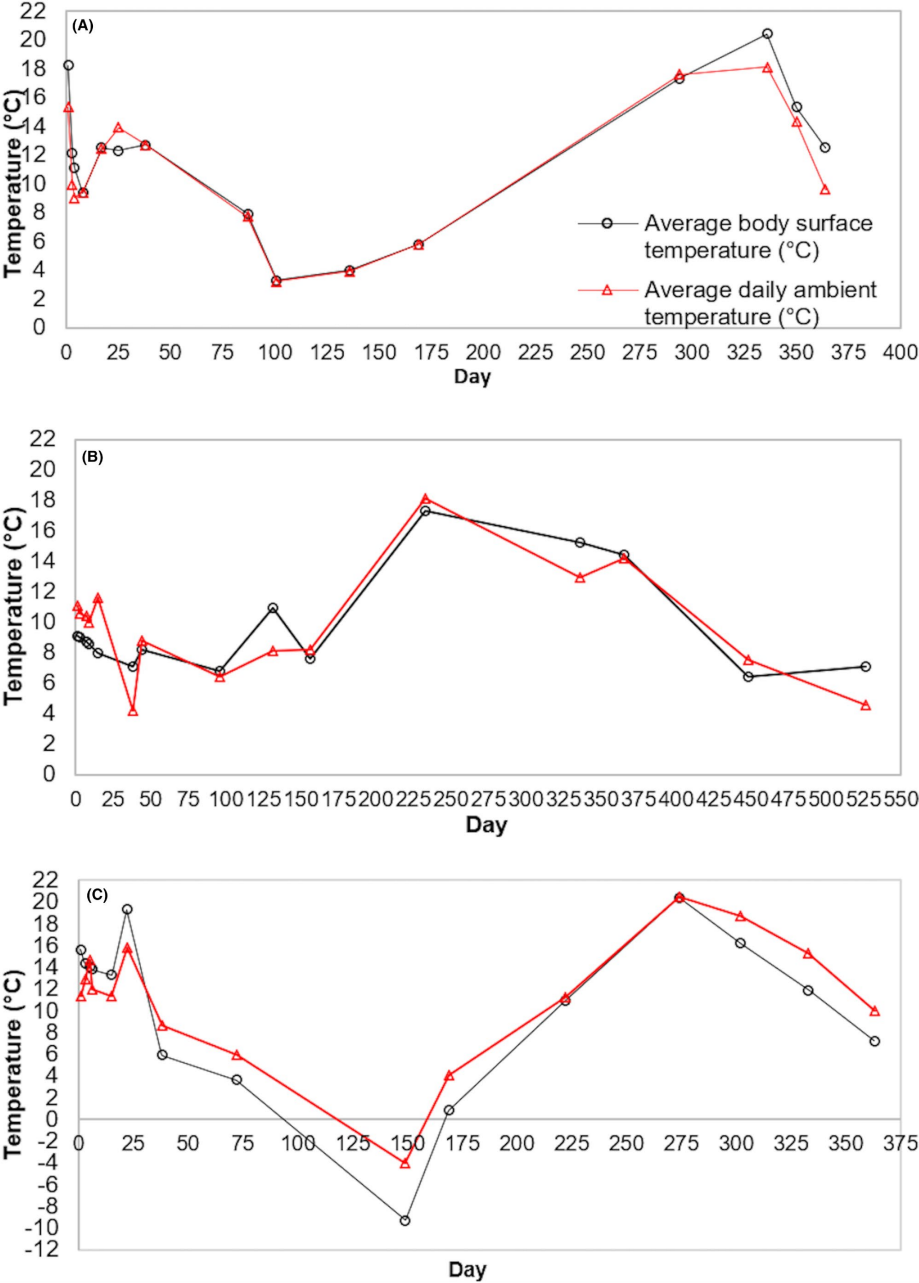
The average body surface temperature of cadavers (°C; black lines and circles) and the average daily ambient temperature (°C; red lines and triangles) on each sampling day for pig cadavers P1 (A), P2 (B), and P3 (C) [Color figure can be viewed at wileyonlinelibrary.com]

The average body surface temperature (°C) reflected the average daily ambient air temperatures (Figure 4A–C, Table S2) with Mann–Whitney *U*‐tests showing that there was no significant difference between the average body surface temperature from cadavers compared to the average daily ambient air temperature of the sampling day (Table 4).

**TABLE 4:** Mann–Whitney *U*‐tests showing no significant difference between the average body surface temperature of all three pig cadavers and the average daily ambient temperature

Average temperature (°C)	*N*	Median	*W*‐Value adjusted for ties	*p*‐Value adjusted for ties
Daily ambient P1	15	9.9	223.50	0.72
Body surface P1	15	12.3		
Daily ambient P2	15	10.0	242.00	0.71
Body surface P2	15	8.6		
Daily ambient P3	15	11.3	231.50	0.98
Body surface P3	15	13.3		

### 3.4 Environmental impacts on mite abundance

**FIGURE 5:**
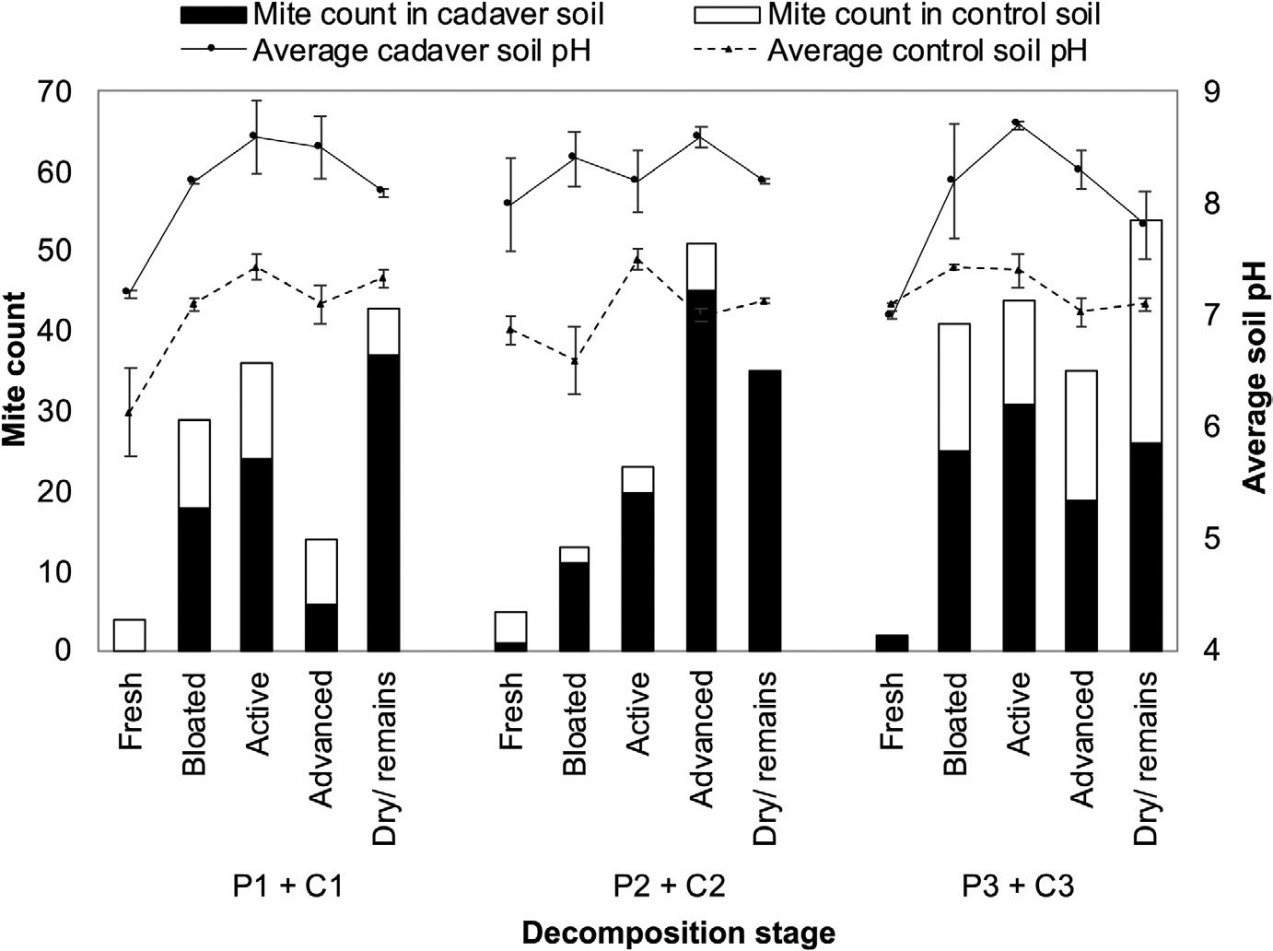
The average pH of soil and mite counts from each pig cadaver (P1, P2, and P3) and the corresponding control soils (C1, C2, and C3) during the stages of decomposition (*X* axis). Error bars represent the standard error (SE) of the means (*n* = 3)

Cadaver decay processes generated significant changes in soil pH (Figure 5). Briefly, fresh cadaver soils and control soils generally exhibited neutral pH (~7), with cadaver soil rapidly becoming significantly more alkaline (~8–9) from the bloated stage onwards. The influence of environmental factors on mite abundance was investigated for cadaver and control soils separately. In cadaver soils, the abundance of mites was found to increase significantly with the increasing soil pH, and there was a significant interaction between the cadaver body temperature and soil pH (Table 5, Table S2). Cadaver soil model fit ($R_{\left(m\right)}^{2}$ = 0.359|$R_{\left(c\right)}^{2}$ = 0.359) indicated that the measured environmental factors, rather than the differences between the replicate cadavers, were responsible for the observed variation in mite abundance.

**TABLE 5:** Results of negative binomial GLMM examining changes in the abundance of cadaver soil mites in response to environmental variables and their two‐way interactions

	Estimate	Std.error	*Z* value	*p* (>[*z*])
(Intercept)	1.923	0.161	11.931	**<2e‐16**
Rel. humidity (%)	−0.038	0.216	−0.177	0.860
Body temp. (°C)	0.091	0.202	0.453	0.650
Rainfall (mm)	0.148	0.215	0.690	0.490
Cad. soil pH	0.408	0.193	2.110	**0.035**
RH*B.temp	0.280	0.193	1.456	0.145
RH*pH	0.318	0.282	1.125	0.260
B.temp*Rain	−0.159	0.259	−0.612	0.541
pH*Rain	0.239	0.308	0.777	0.437
B.temp*pH	0.729	0.261	2.795	**0.005**

Note

Environmental variables were centred and scaled. RH*Rain interaction term removed due to high variance‐inflation factor. In bold, significantly different *p* values.

In control soils, the abundance of mites was not significantly affected by any of the measured environmental variables (air temperature, rainfall, relative humidity and soil pH) (Table 6). Control soil model fit ($R_{\left(m\right)}^{2}$ = 0.033|$R_{\left(c\right)}^{2}$ = 0.102) indicated that the differences between replicates, rather than measured environmental factors, were responsible for most of the observed variation in mite abundance.

**TABLE 6:** Results of generalized linear model with a negative binomial error structure examining changes in the abundance of control soil mites in response to the environmental variables (using replicate as a random factor)

	Estimate	Std.error	*Z* value	*p* (>[*z*])
(Intercept)	0.874	0.303	2.886	**0.004**
Rel. humidity (%)	−0.255	0.198	−1.288	0.198
Air temp. (°C)	−0.064	0.202	−0.317	0.751
Rainfall (mm)	−0.076	0.218	−0.347	0.729
Control soil pH	−0.071	0.243	−0.291	0.771

Note

Environmental variables were centred and scaled. All interaction terms were removed due to high variance‐inflation factors. In bold, significantly different *p* values.

## 4 DISCUSSION

This study demonstrated that cadaveric decay in shallow soil graves follows the same recognized physical postmortem changes associated with the five major stages of decay as reported by previous studies of buried cadavers [5, 21, 40] and surface cadavers [4, 57, 58, 59] in similar terrestrial environments and climatic conditions: fresh, bloated, active, advanced, and dry/remains. We found that the abundance of mites rapidly increased in cadaver soils, with significantly greater numbers of mites in bloated, active, advanced, and dry/remains stages compared to the fresh stage. Mite abundance in cadaver soils was significantly greater than in control soils during the active, advanced, and dry/remains stage, suggesting that the mite abundance alone can be a useful indicator of a shallow grave. The soil pH and the interaction between pH and body temperature, influenced mite abundance in cadaver soils, but no significant association between the environmental factors and mite abundance were detected in control soils.

### 4.1 Decomposition of vertebrate cadavers in shallow graves

Buried bodies are associated with significantly slower decomposition rates mainly due to the inhibited access for necrophagous insects, which along with the lowered temperature, is one of the major factors that results in delays to soft tissue breakdown [11, 21, 26, 60, 61, 62, 63]. In our study, all three cadavers took up to or more than 12 months to transition into full‐body skeletonization, which is similar to the decomposition rates seen in the previous studies of buried cadavers [8, 10, 21, 64].

The decomposition rate of a buried body, especially in shallow graves (<30 cm), can be further influenced by environmental factors such as temperature, humidity, snowfall, and rainfall. Here, no significant differences were observed between the body surface temperatures of the pig cadavers and the daily average ambient temperature for any cadaver. This demonstrated that the temperatures within the graves were closely associated with the ambient temperatures and were directly proportional to the daily and seasonal fluctuations. Hence, slight variations in environmental parameters during each year of the study were likely to have had an influence on the rate of individual decay stages and collectively contributed to the small variations in the decomposition rates between the three cadavers. For example, cadaver P2 transitioned into bloated, active, advanced, and dry/remains later than cadavers P1 and P3, resulting in an overall slower decomposition rate. Despite this, all three cadavers followed similar patterns of duration associated with each decay stage: fresh stages were the shortest stage, followed by the bloated, active, dry, and finally the advanced stage. This pattern conforms to the observations made in the previous studies of buried cadavers [5, 10, 21, 40], exposed cadavers [25, 57, 58], and cadavers concealed via other methods [65].

### 4.2 Changes in mite abundance between decay stages

Mite abundance increased quickly and consistently following the addition of cadavers. Abundance during the fresh stage (~0–7 days) was comparable to the background levels exhibited by the control soils but had increased by the bloated stage (~7–39 days), with a significantly greater abundance of mites sustained in cadaver compared to the control soils from the active stage (~40–153 days) onward through the advanced (~154–333 days) and dry/remains (~334–417 days) stages.

### 4.3 Effect of environmental parameters on decomposition

Cadaver body surface temperature throughout decomposition was directly associated with the ambient temperature, demonstrating that shallow burial appeared to have minimal effect on the grave temperature. Unlike with deeper graves (e.g., >60 cm), cadavers in shallow graves are less sheltered from above the ground environmental fluctuations [10, 23, 60, 66]. Therefore, as with exposed cadavers, seasonal temperature changes are likely to have a greater impact on prolonging individual decay stages of shallowly buried cadavers, particularly during the winter seasons. The rate of autolysis resulting in the formation of putrefactive gases during fresh and bloated stages may have been reduced by the lower seasonal temperatures during the autumn‐winter months (October to November) when all the cadavers were undergoing fresh and bloated decay. The reduced production of gases delays their detection by Diptera and prolongs decomposition.

Active decay is associated with an optimum larval activity and soft tissue breakdown, but low temperatures inhibit microbial activity and decrease the rate of larval development, which may entirely cease below 6 °C, resulting in prolonged soft tissue decay [3, 5, 67]. All three cadavers underwent active decomposition during November to early March when the average monthly ambient temperatures were the lowest for each year of the study. The snowfall occurring in March 2018 may have contributed to slowing down the active decay of cadaver P3 from February to March 2018, which lasted 131 days compared to 98 days for cadaver P1 and 112 days for cadaver P2.

Advanced decay was the most extended stage of decay for all cadavers and was significantly longer than the fresh and bloated stages. A cadaver transition to the advanced decay stage after most of the soft tissue is consumed during the active decay leaving only remnants of flesh, hair, skin, and cartilage [41]. Third instar Dipteran larvae migrate away from the body at this stage and consummation of the remaining flesh and hard tissue slows down. In typical circumstances, the dry/remains stage is the most prolonged stage as bone decomposition may take years and has no definitive endpoint [3]. The low temperatures during February to March 2018 when cadaver P2 was undergoing the dry stage, may explain why the flesh remnants took so long to break down and for the soil, bones, and skin to reach visible desiccation, compared to P1 and P3.

### 4.4 Effect of environmental parameters on mite abundance

A total of 300 mites from cadaver soils and 129 from control soils were covered throughout the decomposition of all three cadavers. The overall lower abundances compared to very high abundances reported earlier in forest soils may be explained by the location of the study site in an urban zone. Urban soils are affected in terms of biodiversity by pollution and environmental disturbances. This in turn can negatively impact the abundance and diversity of soil invertebrates such as mites in urban soils. The soil pH was the only measured environmental parameter that had a significant direct effect on the abundance of mites in cadaver soils. A positive relationship was found, with mite abundance increasing as the soil pH increased. An interactive effect was also observed between the body temperature and the soil pH: when both temperature and pH were low, mite abundance increased, and when both the temperature and the pH were high, mite abundance increased. By contrast, none of the other measured environmental parameters exhibited a significant influence on the mite abundance in control soils, demonstrating that the soil pH is one of the most important factors affecting mite densities in graves.

As decomposition enters bloated and active decay, putrefaction produces a myriad of carbon, nitrogen, and phosphorus‐based volatile organic compounds (VOCs) as by‐products. The mixture of decay fluids, fauna, flora, and highly nutritious VOCs influence the chemical properties of soil, in particular the pH [41]. Cadaver decomposition increases the soil pH because of ammonium (${\text{NH}}_{4}^{+}$) production, which is a by‐product of the catabolism of amino acids [37, 40]. Accumulation of ${\text{NH}}_{4}^{+}$ occurs during mid‐decomposition when the majority of soft tissue breakdown takes place; hence, explaining why there was an increase in the soil pH beneath all three cadavers from relatively neutral during fresh decay to moderately alkaline throughout bloated to advanced decay. A decline in the soil pH toward the very end of the decay (dry/remains stage) was observed from moderately to slightly alkaline. This can be attributed to a decrease in ${\text{NH}}_{4}^{+}$ and other carbon and phosphorus‐based compounds in the soil during the late stages of decomposition after completion of soft tissue decay during the active stage. The increase in the soil pH during early to intermediate stages of decay and a decline during the dry stages has been reported in other decomposition studies of exposed and buried cadavers in various soil types [8, 10, 37, 39, 60, 64, 68].

The soil pH is known to impact densities of bacteria and fungi [46], as well as soil meso‐fauna such as collembola and nematodes, which in turn act as the main prey for predatory soil mites, such as Mesostigmata, which tend to increase in numbers as decomposition progresses [31, 69]. This explains why mite occurrence and abundance were significantly affected by the soil pH during decomposition. Acidic conditions are known to be more favorable for certain mite groups, such as Oribatida mites, while alkaline conditions are less favorable for this group of mites [70].

While the cadaver body temperatures were closely related to the above‐ground temperature fluctuations, the ambient temperature did not have a direct effect on the abundance of mites throughout the decomposition process, only displaying an interaction with cadaver soil pH. Similarly, while fluctuations in the ambient relative humidity, rainfall, and snowfall may have had some impact on delaying the rate of cadaver decay, they did not appear to significantly influence cadaver decay or the abundance of mites throughout the decomposition. This agrees with some previous studies showing that seasonal and environmental fluctuations, such as soil moisture content, did not have a significant effect on the abundance of soil mites [71, 72], but contrasts with other studies that found a significant correlation between mite abundance and soil moisture content [73, 74].

In conclusion, our study show that vertebrate cadavers buried in shallow graves (<30 cm) will undergo the same post‐mortem changes and decomposition stages as previously described for exposed cadavers in terrestrial outdoor environments. However, the rate of decomposition is significantly slower and is influenced by a combination of reduced necrophagous dipteran activity and environmental fluctuations. Cadavers buried in shallow graves cause an increase in the soil pH. Previous studies have demonstrated that knowledge on the abundance of mites colonizing decomposing cadavers in relation to environmental conditions contributes with the information on the stage of decay and estimations of the post mortem interval [75, 76, 77]. This study has demonstrated that an abundance of mites colonizes shallowly buried cadavers, with total mite numbers rapidly increasing between fresh and later stages of decay. We have also shown that fluctuations in the above‐ground temperature, humidity, rainfall, and snowfall impact the overall decomposition rate, while fluctuations of the pH of the grave soil significantly and directly influenced the abundance of mites colonizing each stage of the decay. This last point demonstrates that the increase in soil pH due to decomposition is the major environmental parameter influencing the abundance of mites in shallow graves. The identification of the Acari from this study is being carried out [78], and the analysis of data at order, family, and species level, aims to compare diversity with stages of decomposition as well as body parts in the shallow grave environment.

## Supporting information

Table S1‐S2
