# Supplementary material for: The impact of the decomposition process of shallow graves on soil mite abundance
Source: J Forensic Sci. 2021 Oct 14;67(2):605–18. doi: 10.1111/1556-4029.14906 (PMC9293349; doi:10.1111/1556-4029.14906)
Supplement: Supplementary file 1 — Table S1‐S2 [file JFO-67-605-s001.docx]

SUPPLEMENTARY TABLE S1 Mite numbers by stage of decay and sample site (soil), either pig cadaver soil or control soil [rep = repeat]

|  | **rep** | **stage** | **soil** | **mites** |
| --- | --- | --- | --- | --- |
| 1 | One | a_Fresh | cadaver | 0 |
| 2 | Two | a_Fresh | cadaver | 0 |
| 3 | Three | a_Fresh | cadaver | 0 |
| 4 | One | b_Bloated | cadaver | 3 |
| 5 | Two | b_Bloated | cadaver | 13 |
| 6 | Three | b_Bloated | cadaver | 2 |
| 7 | One | c_Active | cadaver | 19 |
| 8 | Two | c_Active | cadaver | 5 |
| 9 | Three | c_Active | cadaver | 0 |
| 10 | One | d_Advanced | cadaver | 2 |
| 11 | Two | d_Advanced | cadaver | 2 |
| 12 | Three | d_Advanced | cadaver | 2 |
| 13 | One | e_Dry | cadaver | 13 |
| 14 | Two | e_Dry | cadaver | 15 |
| 15 | Three | e_Dry | cadaver | 9 |
| 16 | One | a_Fresh | cadaver | 0 |
| 17 | Two | a_Fresh | cadaver | 1 |
| 18 | Three | a_Fresh | cadaver | 0 |
| 19 | One | b_Bloated | cadaver | 5 |
| 20 | Two | b_Bloated | cadaver | 3 |
| 21 | Three | b_Bloated | cadaver | 3 |
| 22 | One | c_Active | cadaver | 7 |
| 23 | Two | c_Active | cadaver | 11 |
| 24 | Three | c_Active | cadaver | 2 |
| 25 | One | d_Advanced | cadaver | 7 |
| 26 | Two | d_Advanced | cadaver | 23 |
| 27 | Three | d_Advanced | cadaver | 15 |
| 28 | One | e_Dry | cadaver | 10 |
| 29 | Two | e_Dry | cadaver | 6 |
| 30 | Three | e_Dry | cadaver | 19 |
| 31 | One | a_Fresh | cadaver | 2 |
| 32 | Two | a_Fresh | cadaver | 0 |
| 33 | Three | a_Fresh | cadaver | 0 |
| 34 | One | b_Bloated | cadaver | 3 |
| 35 | Two | b_Bloated | cadaver | 14 |
| 36 | Three | b_Bloated | cadaver | 8 |
| 37 | One | c_Active | cadaver | 13 |
| 38 | Two | c_Active | cadaver | 17 |
| 39 | Three | c_Active | cadaver | 1 |
| 40 | One | d_Advanced | cadaver | 7 |
| 41 | Two | d_Advanced | cadaver | 11 |
| 42 | Three | d_Advanced | cadaver | 1 |
| 43 | One | e_Dry | cadaver | 6 |
| 44 | Two | e_Dry | cadaver | 16 |
| 45 | Three | e_Dry | cadaver | 4 |
| 46 | One | a_Fresh | control | 1 |
| 47 | Two | a_Fresh | control | 1 |
| 48 | Three | a_Fresh | control | 2 |
| 49 | One | b_Bloated | control | 2 |
| 50 | Two | b_Bloated | control | 8 |
| 51 | Three | b_Bloated | control | 1 |
| 52 | One | c_Active | control | 3 |
| 53 | Two | c_Active | control | 7 |
| 54 | Three | c_Active | control | 2 |
| 55 | One | d_Advanced | control | 0 |
| 56 | Two | d_Advanced | control | 7 |
| 57 | Three | d_Advanced | control | 1 |
| 58 | One | e_Dry | control | 0 |
| 59 | Two | e_Dry | control | 1 |
| 60 | Three | e_Dry | control | 5 |
| 61 | One | a_Fresh | control | 2 |
| 62 | Two | a_Fresh | control | 1 |
| 63 | Three | a_Fresh | control | 1 |
| 64 | One | b_Bloated | control | 0 |
| 65 | Two | b_Bloated | control | 2 |
| 66 | Three | b_Bloated | control | 0 |
| 67 | One | c_Active | control | 2 |
| 68 | Two | c_Active | control | 1 |
| 69 | Three | c_Active | control | 0 |
| 70 | One | d_Advanced | control | 1 |
| 71 | Two | d_Advanced | control | 1 |
| 72 | Three | d_Advanced | control | 4 |
| 73 | One | e_Dry | control | 0 |
| 74 | Two | e_Dry | control | 0 |
| 75 | Three | e_Dry | control | 0 |
| 76 | One | a_Fresh | control | 0 |
| 77 | Two | a_Fresh | control | 0 |
| 78 | Three | a_Fresh | control | 0 |
| 79 | One | b_Bloated | control | 7 |
| 80 | Two | b_Bloated | control | 2 |
| 81 | Three | b_Bloated | control | 7 |
| 82 | One | c_Active | control | 3 |
| 83 | Two | c_Active | control | 6 |
| 84 | Three | c_Active | control | 4 |
| 85 | One | d_Advanced | control | 1 |
| 86 | Two | d_Advanced | control | 1 |
| 87 | Three | d_Advanced | control | 14 |
| 88 | One | e_Dry | control | 1 |
| 89 | Two | e_Dry | control | 4 |
| 90 | Three | e_Dry | control | 23 |

SUPPLEMENTARY TABLE S2 Data of environmental parameters (above ground), pH and body temperature (in soil), and mite numbers by carcass and control [key found at the bottom of the table]

|  | **cadaver** | | **stage** | **tempAir** | | **RH** | | **Rain** | | **Snow** | | **pH_Pig** | | **pH_Con** | | **tempBody** | | **MitesPig** | | **MitesCon** | |
| --- | --- | --- | --- | --- | --- | --- | --- | --- | --- | --- | --- | --- | --- | --- | --- | --- | --- | --- | --- | --- | --- |
| 1 | One | a_Fresh | | | 15.3 | 96 | 7.1 | | 0 | | 7.3 | | 5.6 | | 18.2 | | 0 | | 1 | |  |
| 2 | One | a_Fresh | | | 9.9 | 87 | 0.1 | | 0 | | 7.2 | | 5.9 | | 12.1 | | 0 | | 1 | |  |
| 3 | One | a_Fresh | | | 9 | 87 | 0 | | 0 | | 7.2 | | 6.9 | | 11.1 | | 0 | | 2 | |  |
| 4 | One | b_Bloated | | | 9.4 | 77 | 0 | | 0 | | 8.1 | | 6.6 | | 9.4 | | 3 | | 2 | |  |
| 5 | One | b_Bloated | | | 12.4 | 75 | 0 | | 0 | | 8.2 | | 7 | | 12.5 | | 13 | | 8 | |  |
| 6 | One | b_Bloated | | | 13.9 | 92 | 0.5 | | 0 | | 8.2 | | 7 | | 12.3 | | 2 | | 1 | |  |
| 7 | One | c_Active | | | 12.7 | 90 | 1.8 | | 0 | | 8 | | 7.1 | | 12.7 | | 19 | | 3 | |  |
| 8 | One | c_Active | | | 7.7 | 78 | 2.8 | | 0 | | 8.8 | | 7.1 | | 7.9 | | 5 | | 7 | |  |
| 9 | One | c_Active | | | 3.2 | 77 | 0.1 | | 0 | | 9.1 | | 7.1 | | 3.3 | | 0 | | 2 | |  |
| 10 | One | d_Advanced | | | 3.9 | 79 | 0.1 | | 0 | | 9.1 | | 7.2 | | 4 | | 2 | | 0 | |  |
| 11 | One | d_Advanced | | | 5.8 | 76 | 0 | | 0 | | 8.3 | | 7.1 | | 5.8 | | 2 | | 7 | |  |
| 12 | One | d_Advanced | | | 17.6 | 69 | 0 | | 0 | | 8.2 | | 7 | | 17.3 | | 2 | | 1 | |  |
| 13 | One | e_Dry | | | 18.1 | 92 | 0.2 | | 0 | | 8 | | 7 | | 20.4 | | 13 | | 0 | |  |
| 14 | One | e_Dry | | | 14.3 | 91 | 4.1 | | 0 | | 8.1 | | 6.8 | | 15.3 | | 15 | | 1 | |  |
| 15 | One | e_Dry | | | 9.6 | 80 | 0 | | 0 | | 8.1 | | 6 | | 12.5 | | 9 | | 5 | |  |
| 16 | Two | a_Fresh | | | 11.1 | 86 | 1.3 | | 0 | | 7.2 | | 7.4 | | 9.1 | | 0 | | 2 | |  |
| 17 | Two | a_Fresh | | | 10.6 | 82 | 0.1 | | 0 | | 8.5 | | 7.5 | | 9 | | 1 | | 1 | |  |
| 18 | Two | a_Fresh | | | 10.4 | 75 | 0.1 | | 0 | | 8.4 | | 7.4 | | 8.7 | | 0 | | 1 | |  |
| 19 | Two | b_Bloated | | | 10 | 83 | 0.1 | | 0 | | 8.7 | | 7.2 | | 8.6 | | 5 | | 0 | |  |
| 20 | Two | b_Bloated | | | 11.6 | 93 | 0.1 | | 0 | | 8.5 | | 7.5 | | 8 | | 3 | | 2 | |  |
| 21 | Two | b_Bloated | | | 4.2 | 85 | 0.1 | | 0 | | 7.9 | | 7.6 | | 7.1 | | 3 | | 0 | |  |
| 22 | Two | c_Active | | | 8.8 | 82 | 0 | | 0 | | 7.9 | | 7.7 | | 8.2 | | 7 | | 2 | |  |
| 23 | Two | c_Active | | | 6.4 | 98 | 10.9 | | 0 | | 8 | | 7.4 | | 6.8 | | 11 | | 1 | |  |
| 24 | Two | c_Active | | | 8.1 | 87 | 0.1 | | 0 | | 8.8 | | 7.4 | | 10.9 | | 2 | | 0 | |  |
| 25 | Two | d_Advanced | | | 8.2 | 91 | 0 | | 0 | | 8.8 | | 7.7 | | 7.6 | | 7 | | 1 | |  |
| 26 | Two | d_Advanced | | | 18.1 | 70 | 0 | | 0 | | 8.6 | | 7.3 | | 17.3 | | 23 | | 1 | |  |
| 27 | Two | d_Advanced | | | 12.9 | 84 | 2.9 | | 0 | | 8.5 | | 7.2 | | 15.2 | | 15 | | 4 | |  |
| 28 | Two | e_Dry | | | 14.2 | 85 | 3 | | 0 | | 8.2 | | 6.8 | | 14.4 | | 10 | | 0 | |  |
| 29 | Two | e_Dry | | | 7.5 | 90 | 10.5 | | 0 | | 8.2 | | 7.1 | | 6.4 | | 6 | | 0 | |  |
| 30 | Two | e_Dry | | | 4.6 | 75 | 0 | | 0 | | 8.1 | | 7.4 | | 7.1 | | 19 | | 0 | |  |
| 31 | Three | a_Fresh | | | 11.3 | 74 | 0 | | 0 | | 7.1 | | 7 | | 15.6 | | 2 | | 0 | |  |
| 32 | Three | a_Fresh | | | 12.9 | 62 | 0 | | 0 | | 7 | | 7.1 | | 14.3 | | 0 | | 0 | |  |
| 33 | Three | a_Fresh | | | 14.7 | 80 | 0.1 | | 0 | | 7 | | 6.9 | | 14 | | 0 | | 0 | |  |
| 34 | Three | b_Bloated | | | 12 | 85 | 0 | | 0 | | 7.2 | | 6.9 | | 13.8 | | 3 | | 7 | |  |
| 35 | Three | b_Bloated | | | 11.3 | 83 | 2.3 | | 0 | | 8.8 | | 7.3 | | 13.3 | | 14 | | 2 | |  |
| 36 | Three | b_Bloated | | | 15.8 | 90 | 0.1 | | 0 | | 8.7 | | 6.9 | | 19.3 | | 8 | | 7 | |  |
| 37 | Three | c_Active | | | 8.6 | 89 | 0.1 | | 0 | | 8.7 | | 7.2 | | 5.9 | | 13 | | 3 | |  |
| 38 | Three | c_Active | | | 5.9 | 90 | 7 | | 0 | | 8.7 | | 7.3 | | 3.6 | | 17 | | 6 | |  |
| 39 | Three | c_Active | | | -4 | 77 | 2.5 | | 1 | | 8.6 | | 7.5 | | -9.3 | | 1 | | 4 | |  |
| 40 | Three | d_Advanced | | | 4.1 | 69 | 0 | | 0 | | 8.5 | | 7.1 | | 0.8 | | 7 | | 1 | |  |
| 41 | Three | d_Advanced | | | 11.2 | 88 | 8.8 | | 0 | | 8.5 | | 7.2 | | 10.9 | | 11 | | 1 | |  |
| 42 | Three | d_Advanced | | | 20.5 | 44 | 0 | | 0 | | 8 | | 7.1 | | 20.4 | | 1 | | 14 | |  |
| 43 | Three | e_Dry | | | 18.7 | 65 | 0 | | 0 | | 8.2 | | 7.1 | | 16.2 | | 6 | | 1 | |  |
| 44 | Three | e_Dry | | | 15.3 | 74 | 0 | | 0 | | 7.2 | | 7.2 | | 11.9 | | 16 | | 4 | |  |
| 45 | Three | e_Dry | | | 10 | 75 | 0 | | 0 | | 8 | | 7 | | 7.2 | | 4 | | 23 | |  |

Stage = stage of decay; tempAir = air temperature; RH = relative humidity; pH_Con/Pig = pH measured at Control or Pig site; MitesPig/Con = mite numbers at control/pig site
